# The Diagnostic Role of Adiponectin in Pulmonary Embolism

**DOI:** 10.1155/2016/6121056

**Published:** 2016-03-06

**Authors:** Evrim Gul, Yeliz Gul, Ersin Yıldırım, Mustafa Safa Pepele, Mustafa Yıldız, Mehmet Nuri Bozdemir, Mehmet Ruhi Onur, Bengü Mutlu, Feti Yıldız, Ömer Doğan Alataş, Necip İlhan

**Affiliations:** ^1^Department of Emergency Medicine, Elazig Education and Research Hospital, 23200 Elazıg, Turkey; ^2^Department of Radiology, Elazig Education and Research Hospital, 23200 Elazıg, Turkey; ^3^Department of Cardiology, Elazig Education and Research Hospital, 23200 Elazıg, Turkey; ^4^Department of Emergency Medicine, Firat University, Elazıg, Turkey; ^5^Department of Radiology, Firat University, Elazıg, Turkey; ^6^Department of Biochemistry, Firat University, Elazıg, Turkey

## Abstract

*Background and Aims*. Pulmonary thromboembolism (PTE) is a frequent disease with difficult diagnosis and high mortality. Misdiagnosis occurs in 2/3 patients and mortality rates reach up to 30%. The aim of our study was to investigate the role of adiponectin used in emergency service in diagnosis of PTE.* Materials and Methods*. 95 patients with suspected PTE included in the study. Plasma adiponectin and D-dimer levels were measured and chest X-ray and multidetector row computed tomography scan obtained. Diagnosis was supported by vascular filling defect on tomography. Control group consisted of patients with suspected PTE and normal chest computed tomography findings.* Results*. Mean D-dimer level was 4241.66 ± 1082.98 ng/mL in patients and 2211.21 ± 1765.53 ng/mL in the control group (*p* ≤ 0.05). Mean adiponectin level was 5.46 ± 4.39 *μ*g/mL in patients and 7.68 ± 4.67 *μ*g/mL in the control group (*p* ≤ 0.05). Wells and Geneva scores were higher in patients compared to the control group.* Conclusions*. As a result, we conclude that lower adiponectin levels have an important role in the diagnosis of PTE.

## 1. Introduction

Pulmonary thromboembolism (PTE) refers to the migration of a blood clot from systemic deep veins into the pulmonary vascular bed. It is a common occurrence with high mortality rates and presents itself as being difficult to diagnose [1]. No precise diagnosis has been provided in approximately 2/3 of the patients experiencing pulmonary thromboembolism and mortality rates of these patients have reached up to 30%. In cases where pulmonary thromboembolism is diagnosed accurately and proper treatment is provided, the mortality rate is likely to decrease by 3% [2]. Thus, physicians and protective health care planners should be aware of the rapid progress in epidemiology, pathophysiology, diagnosis, treatment, and protection strategies of PTE.

Today there is no biomarker for the diagnosis of pulmonary embolism specifically recommended in the guidelines. To introduce the latest guidelines D-dimer has been mentioned but from positive predictive value of elevated D-dimer levels is low and D-dimer testing is not useful for confirmation of pulmonary embolism. There is no standard for the diagnosis of pulmonary embolism. Some prediction rules have been developed, such as wells and Geneva scores. Both have been adequately validated and are used in daily practice. But today, despite all these rules many patients with pulmonary embolism cannot be diagnosed. Therefore we need new biomarkers that may help in the diagnosis.

Adiponectin, being an adipose tissue protein, accounts for 0.01% of human plasma proteins. Adiponectin is a glycoprotein containing 244 amino acids [3] and it has a significant role in regulating glucose and lipid metabolism over insulin-sensitive tissues in both humans and animals [3, 4]. The protective role of adiponectin in patients at high-risk for recurrent cardiovascular disease such as metabolic syndrome has been proven. High adiponectin levels have an effect on decreasing mortality in all cardiovascular diseases [5]. Experimental studies have indicated that adiponectin has potential antiatherogenic and anti-inflammatory properties [6–8]. In* in vivo* studies conducted in human aortic endothelial cells, it has been shown that adiponectin in adhesion molecules regulating vascular inflammatory response leads to a dose-dependent reduction [9]. Adiponectin accumulates in the atherosclerotic vein wall in a dose-dependent manner and inhibits the cell migration induced by TNF-*α* [10]. It has been shown that hypoadiponectinemia in the carotid artery and arterial walls is associated with thickening of the intima and media layers [5].

The purpose of the present study was to investigate the relationship between serum adiponectin level and PTE.

## 2. Materials and Methods

After approval from the Ethics Committee, 95 patients hospitalized and monitored with PTE diagnosis were enrolled in the study conducted between January 2009 and May 2010 in the Emergency Medicine Clinic of Fırat University Hospital. Written informed consents of all patients agreeing to take part in the study were also taken. The diagnosis of pulmonary thromboembolism was made based on its compatibility with filling defect of PTE on multidetector computed tomography (MDCT) according to predefined standard protocol. The control group involved patients presenting to the emergency department with a suspicion of pulmonary embolism, yet this could not be detected on a Thorax CT scan.

### 2.1. Biochemical Analysis

D-dimer levels, which were drained into EDTA tubes, were measured in the emergency department via equipment called Biosite Triage Meter Plus® (San Diego, USA). The cutoff value was determined as 500 ng/mL. 5 mL of venous blood sample was drawn from all cases for adiponectin measurement. After allowing the blood to clot for 30 minutes, the blood was then separated into serum by centrifugation for 3 minutes at 5000 g. The separated serums were kept at −20°C until analysis time for adiponectin level. Adiponectin was analyzed via Assay Max Human adiponectin (Acrp30) ELISA kit® (ASSAYPRO®, 41 Triad South Drive, St. Charles, MO 63304, USA) through ELISA (enzyme-linked immunosorbent inc.) method. The results were recorded as *μ*g/mL.

### 2.2. Statistical Analysis

In the study, data obtained were indicated as mean ± standard deviation. For statistics, the statistical package SPSS 11.00 (SPSS Inc.®, Software Chicago, IL, USA) was used. While Student's *t*-test was used for the analysis of continuous variables, chi-square test was utilized for the analysis of categorical data. Correlation analysis was made by Pearson correlation analysis method. A *p* value ≤0.05 was considered statistically significant. Statistical power of the study was calculated by 75%.

## 3. Results

A total of 95 patients, of whom 58 (61.1%) were females and 37 (38.9%) were males, were included in the study. Of 38 patients diagnosed with PTE, 24 were females and 14 were males. No statistical difference was detected between patients in terms of gender (*p* > 0.05).

Mean age of the patients was 59.83 ± 17.32 years. While average age of female patients was 60.05 ± 18.38, for the male patients it was 59.48 ± 15.76 years. In the overall evaluation, no statistical difference in terms of mean age was found (*p* > 0.05).

Clinically, the average heart rate in the patient group with PTE was measured 105.97 ± 21.20 beats/min.; in the non-patient group it was 99.79 ± 21.69 beats/min. (*p* > 0.05). Information regarding Wells and Geneva scores was shown in Table 1.

The Wells score used in the diagnosis of pulmonary thromboembolism was found as 5.04 ± 1.95 in the patient group with PTE, whereas it was 4.07 ± 1.90 (*p* ≤ 0.05) in the control group. While the Geneva score was indicated as 7.30 ± 2.39 in the patient group with PTE, in the control group it was measured as 6.03 ± 2.08 and the difference between these two groups was statistically significant (*p* ≤ 0.05).

Anatomically the most frequently occluded segment was the main pulmonary artery with 18.9%, followed by right pulmonary artery and its branches with 14.9% and left pulmonary artery and its branches with 6.4%.

The hospital stay of the patient group with PTE in the emergency department was measured as 185.79 ± 10.99 sec; however, in the control group, it was found 223.86 ± 13.53 sec and the difference was statistically significant (*p* ≤ 0.05). Only 2 of the patients with PTE required intensive care treatment. Four patients (4.2%) died.

D-dimer level of all patient group included in the study was found as 3003.27 ± 1832.19 ng/mL and in the patient group with PTE diagnosis it was 4241.66 ± 1082.98 ng/mL; in the control group it was measured as being 2211.21 ± 1765.53 ng/mL and this finding was statistically significant (*p* ≤ 0.05). D-dimer level in all patients with PTE diagnosis was found high. In none of the five patients with normal D-dimer level, PTE was detected. Negative predictive value (NPV) was indicated as 100%.

In all patient groups, mean adiponectin level was measured 6.70 ± 4.59 *μ*g/mL, in the patient group with PTE diagnosis it was 5.46 ± 4.39 *μ*g/mL and in the control group it was 7.68 ± 4.67 *μ*g/mL; a statistically significant difference was detected between the two groups (*p* ≤ 0.05). In the correlation analysis, adiponectin levels were significantly correlated with systolic blood pressure, Geneva score, and BNP level (Table 2).

## 4. Discussion

Pulmonary thromboembolism presents itself as a vital clinical problem which emerges in the form of a blockage in pulmonary artery branches due to an embolus that originates mostly from a venous system. The importance of PTE results from its high mortality and preventable nature, difficulty in diagnosis, and, lastly, difficulty in determining various treatment modalities for the patients already diagnosed. Strategies in diagnosis and treatment vary in terms of clinical manifestations and existence of hemodynamic disorder [11].

Laboratory findings of pulmonary thromboembolism are nonspecific. The most useful laboratory parameter for emergency departments is the D-dimer level. In a study by Perrier et al. [12], while the normal plasma level of D-dimer was eliminated by ELISA, PTE was excluded by nondiagnostic V/Q scintigraphy results. In our study, the D-dimer level of all patient groups with PTE was found high. The D-dimer level in the patient group was significantly higher than the control group (*p* ≤ 0.05). In 5 out of 95 patients who were included in the study, D-dimer level was normal and on none of these patients' MDCT, PTE was detected. About this finding, NPV was indicated as 100%. Regardless of the test method, patients with high D-dimer level must undergo further examinations in clinically suspicious cases. A negative D-dimer result reliably excludes PTE diagnosis in patients with low and intermediate clinical probability of pulmonary embolism. Although the value of a positive D-dimer test is not specific, a negative D-dimer test is quite a reliable parameter in excluding PTE. A negative result in routinely used D-dimer test is treated very sensitively in excluding PTE diagnosis in cases with low clinical suspicion. However, negative D-dimer should not be predictive in excluding the diagnosis in cases with high clinical suspicion, and further examinations should be performed [13, 14]. We believe that the assessment of D-dimer level is a noninvasive subsidiary method in excluding PTE in patients with low and intermediate clinical suspicion and should particularly be used in emergency department settings.

Today, there are new biomarkers that can be used in the diagnosis of pulmonary embolism and are being investigated. In the new studies, serum copeptin and plasma miRNA-28-3p levels have been shown to be useful in the diagnosis of pulmonary embolism [15, 16]. In previous studies, MMP-9 and PAI-1 levels have been shown to be beneficial for the diagnosis of suspected pulmonary embolism [17]. In addition, the increased MMP levels have been shown to be associated with lung tissue damage and it was shown that MMP inhibition with doxycycline protects against acute pulmonary embolism-induced mortality and RV enlargement [18].

It has been determined that adipocytes do not only serve as essential tissues in storing fats but also play crucial key roles in controlling energy and homeostasis in metabolic and inflammatory signals. The effects of adiponectin in the body are as follows [19]: it increases insulin sensitivity; improves lipid level; acts as an anti-inflammatory agent; shows antiatherosclerotic effect; regulates angiogenic and endothelial function; and has antiapoptotic impact. Experimental studies have suggested that adiponectin has potential antiatherogenic and anti-inflammatory properties. In the studies regarding human aortic cells, it has been shown that adiponectin leads to a dose-dependent decrease in adhesion molecules, which regulate vascular inflammatory response [8, 9]. In some experimental animal models, it has been demonstrated that adiponectin may play a protective role against atherosclerotic agents due to its anti-inflammatory effect [20]. Matsuda et al. showed that when damage was done by a catheter in the vein wall of mice, which lack adiponectin, the veins of smooth muscle cells of these mice result in neointimal thickening by proliferation. They also reported that when the same mice were supplemented with adiponectin recombinant adenovirus, neointimal proliferation in the damaged arterial wall improved. They proved that increased serum adiponectin levels in APN-KO mice reduced significantly the progression of atherosclerotic lesions [21]. Hotta et al. demonstrated that serum adiponectin level was lower in diabetics with coronary artery disease than the diabetics without coronary artery diseases. It has also been reported in this study that serum adiponectin concentrations are negatively correlated with serum glucose, insulin, and triglyceride levels but positively correlated with serum HDL cholesterol levels [22]. In the clinical study by Bang et al. (Bang, 2007 #3) including 231 patients, serum adiponectin level was found lowest in the group with intracranial atherosclerosis, highest in the cardioembolic group, and medium-low in the extracranial atherosclerosis and small artery blockage [23].

Here, we endeavored to find answers for the questions that present the most important part of our study, “does adiponectin have a role in reducing mortality and morbidity in the early diagnosis and treatment of PTE?” “Can serum adiponectin level, therefore, be considered as a risk factor?” With regard to our findings, when we analyzed serum adiponectin levels of a total of 95 patients, we indicated a statistically significant difference between 38 patients diagnosed with PTE and 57 patients who were not diagnosed with PTE (*p* ≤ 0.05). Adiponectin levels in the patient group with PTE were significantly low and these findings have an important role in the diagnosis of PTE.

## 5. Conclusion

As a result, we conclude that lower adiponectin levels have an important role in the diagnosis of PTE. Further studies are needed to support the findings so that patients with pulmonary embolism can receive accurate and immediate diagnosis in emergency departments.

## Figures and Tables

**Table 1:** Baseline characteristics in the study groups.

	Control group (*n* = 57)	PTE group (*n* = 38)	*p*
Age (years)	59.48 ± 15.76	60.05 ± 18.38	NS
Sex (male/female)	23/34	14/24	NS
Heart rate (beat/min)	99.79 ± 21.69	105.97 ± 21.20	NS
Wells score	4.07 ± 1.90	5.04 ± 1.95	<0.05
Geneva score	6.03 ± 2.08	7.30 ± 2.39	<0.05
D-dimer (ng/mL)	2211.21 ± 1765.53	4241.66 ± 1082.98	<0.05
Adiponectin (*μ*g/mL)	7.68 ± 4.67	5.46 ± 4.39	<0.05
WBC count (10^3^/*μ*L)	6.8 ± 1.9	7.1 ± 2.3	NS
Platelet count (10^3^/*μ*L)	228 ± 61	242 ± 73	NS
Hemoglobin (g/dL)	13.8 ± 1.3	13.5 ± 1.6	NS

PTE: pulmonary thromboembolism; *p* ≤ 0.05 was accepted as statistically significant.

Wells score clinical probability: 0-1: low, 2–6: intermediate, >7: high.

Geneva score clinical probability: 0–3: low, 4–10: intermediate, >11: high.

**Table 2:** The correlation analysis of the adiponectin.

Parameters	*r*	*p*
Adiponectin		
Systolic BP	−0.368	≤0.05
Geneva score	0.385	≤0.05
BNP	0.471	≤0.05

BNP: brain natriureticpeptide; BP: blood pressure; *p* ≤ 0.05 was accepted as statistically significant.
